# Analysis of KRAS, NRAS and BRAF mutational profile by combination of in-tube hybridization and universal tag-microarray in tumor tissue and plasma of colorectal cancer patients

**DOI:** 10.1371/journal.pone.0207876

**Published:** 2018-12-18

**Authors:** Francesco Damin, Silvia Galbiati, Nadia Soriani, Valentina Burgio, Monica Ronzoni, Maurizio Ferrari, Marcella Chiari

**Affiliations:** 1 Istituto di Chimica del Riconoscimento Molecolare, CNR, Milano, Italy; 2 Unit of Genomic for the Diagnosis of Human Pathologies, Division of Genetics and Cell Biology, IRCCS San Raffaele Scientific Institute, Milan, Italy; 3 Dipartimento di Oncologia Medica, IRCCS Ospedale San Raffaele, Milan, Italy; 4 Laboratory of Clinical Molecular Biology, IRCCS Ospedale San Raffaele, Milan, Italy; 5 Università Vita-Salute San Raffaele, Milan, Italy; University of Nebraska Medical Center, UNITED STATES

## Abstract

Microarray technology fails in detecting point mutations present in a small fraction of cells from heterogeneous tissue samples or in plasma in a background of wild-type cell-free circulating tumor DNA (ctDNA). The aim of this study is to overcome the lack of sensitivity and specificity of current microarray approaches introducing a rapid and sensitive microarray-based assay for the multiplex detection of minority mutations of oncogenes (*KRAS*, *NRAS* and *BRAF***)** with relevant diagnostics implications in tissue biopsies and plasma samples in metastatic colorectal cancer patients. In our approach, either wild-type or mutated PCR fragments are hybridized in solution, in a temperature gradient, with a set of reporters with a 5' domain, complementary to the target sequences and a 3' domain complementary to a surface immobilized probe. Upon specific hybridization in solution, which occurs specifically thanks to the temperature gradients, wild-type and mutated samples are captured at specific location on the surface by hybridization of the 3’ reporter domain with its complementary immobilized probe sequence. The most common mutations in *KRAS*, *NRAS* and *BRAF* genes were detected in less than 90 minutes in tissue biopsies and plasma samples of metastatic colorectal cancer patients. Moreover, the method was able to reveal mutant alleles representing less than 0,3% of total DNA. We demonstrated detection limits superior to those provided by many current technologies in the detection of *RAS* and *BRAF* gene superfamily mutations, a level of sensitivity compatible with the analysis of cell free circulating tumor DNA in liquid biopsy.

## Introduction

The identification of DNA variants that can cause diseases is a central aim in human genetics. In particular, the ability to detect mutations in oncogenes facilitates early diagnosis, monitoring and treatment [1,2] of cancer. The discovery that tumor cells release DNA fragments (circulating tumor DNA -ctDNA-) in blood, urine or other body fluid samples, paves the way to a paradigm shift in cancer diagnostics introducing the concept of liquid biopsy: a term that refers to a novel, non-invasive technique used for detecting cancer biomarkers [3,4]. ctDNA belongs to the pool of the total circulating cell free-DNA in blood. The mechanisms of its release are not completely disclosed; probably it derives from apoptotic or necrotic cells as well as from living cells through a mechanism of active secretion. ctDNA provides real-time molecular information allowing monitoring treatment response and relapsing as it contains genetic alteration of both primary and metastatic lesions, such as point mutations, copy number variations and insertions/deletions [5,6].

Detecting mutation in ctDNA is challenging since the lower number of mutant copies of cancer origin are masked by the large amount of wild-type DNA mostly from contaminant leukocytes [7]. Liquid biopsy is still in its infancy and efforts will be required before the field can mature and achieve widespread routine use in oncology clinical practice. The analysis of low-abundance mutations requires cfDNA isolation and amplification followed by mutations detection either in disease specific genes (PCR based sequencing) [8–12] or in multiple genes simultaneously (next generation sequencing -NGS- multiplex testing) [13]. Droplet digital PCR (ddPCR) is one of newly developed methods that allow for enumeration of rare mutant variants. Based on water-emulsion droplet technology, ddPCR fractionates a DNA sample in 20.000 droplets [14]. Mutation-specific amplification of the template subsequently occurs in each individual droplet, and counting the positive droplets gives precise, absolute target quantification as copies per milliliter of plasma. It was reported that ddPCR can detect mutant alleles with high sensitivity (0.01–0.001%) [15]. However, with ddPCR only the genes that are the most susceptible to mutations are analyzed, at first, giving the patient the choice of whether to pursue additional tests based on the results. The downside of this approach to testing is it is time and cost consuming.

Another sophisticated ctDNA based cancer test is the targeted amplicon sequencing [16,17]. NGS in particular conditions, can reach the high sensitivity required for the analysis of ctDNA. This technique has the potential to uncover additional actionable findings that could have otherwise gone undetected by the traditional single gene serial testing but it is expensive and time-consuming. Moreover, it allows to process in parallel only a limited number of samples and demands bioinformatics skills or already developed bioinformatics tools specific for plasma samples.

In this study, in an effort to overcome the limitations of both existing approaches, we introduce an innovative assay for the simultaneous detection of single mutations in different oncogenes, based on microarray technology combined with solution hybridization. DNA microarrays have been used for years in genotyping applications, including SNP typing. Affymetrix and Illumina commercialize SNP array platforms for the genotyping of millions of SNPs [18]. However, to the best of our knowledge, there are no examples of mutation detection in liquid biopsy by microarray as none of the methods reported in the literature meets the sensitivity and specificity requirements of liquid biopsy. In this work, we hybridize single stranded tagged-PCR products with dual-domain oligonucleotide reporters. The domain sequences complementary to regions encompassing the mutation form hybrids in solution that are direct by the second domain to specific locations on the microarray surface. The tagged-PCRs captured on the microarray are revealed by a fluorescent universal oligonucleotide.

The method was validated in mutation detection of the *RAS* proto-oncogene superfamily (in particular *KRAS* and *NRAS*) and *BRAF* [19–21].

In colorectal cancer (CRC) approximately 30% to 50% of tumors have *KRAS* mutations that occur early in the tumorigenesis pathway, so the detection of *KRAS* mutations is useful for early diagnosis, prognosis and evaluation of the therapeutic outcome in cancer treatment [22]. *KRAS* is an effector molecule of epidermal growth factor receptor (*EGFR*), a key target of therapeutic strategies designed to treat metastatic CRC. Constitutively, activating mutations at codon 12, 13, 61 or 146 can determine resistance to *EGFR*-target therapies and patients harboring such mutations do not receive benefit from anti-*EGFR* treatment. Also *NRAS* mutation that is found in less than 5% of CRC patients [23,24], may affect the efficacy of anti-*EGFR* antibodies [25]. There is also increasing evidence that B-Raf proto-oncogene (*BRAF*) mutations, which confer a worse prognosis, also determine a higher degree of resistance [26]. The National Comprehensive Cancer Network (NCCN) and the American Society for Clinical Oncology (ASCO) have recently published guidance recommending testing of exon 2 (codons 12 and 13), 3 (codon 61), and 4 (codon 146) in both *KRAS* and *NRAS* genes [27] prior to anti-*EGFR* therapy. In spite of the importance to genotype with high accuracy the hot-spot regions of *RAS* family gene, only very few commercially available tests cover all these codons at the same time.

With this multiplex system, we were able to correctly genotype simultaneously the most frequent KRAS mutations in exon 2 (codon 12 and 13), 3 (codon 61) and 4 (codon 146), *NRAS* mutations in exon 2 (codon 12 and 13) and *BRAF* mutation in exon 15 (V600E) with high sensitivity (less than 0,3% of mutant DNA) on both, tissue and liquid biopsy samples. Our results demonstrate the applicability of the method for routine diagnosis of cancer in in clinical practice.

## Materials and methods

### Samples

To prove the assay specificity of all the 22 mutations analyzed that is *KRAS* codon 12 (G12A, G12C, G12D, G12R, G12S, G12V), 13 (G13D), 61 (Q61H - A>C and A>T-, Q61K, Q61L, Q61R) and 146 (A146T), *NRAS* codon 12 (G12A, G12C, G12D, G12S, G12V) and 13 (G13D, G13R, G13V) and *BRAF* codon 15 (V600E), we used the heterozygous reference standards by Diatech Pharmacogenetics with the exception of the mutations KRAS Q61R, Q61H (A>T), Q61K, A146T and NRAS G12A, G12C, G12S, G12V, G13R and G13V where the references were not available. Thus, mutagenized DNA containing alternatively all the considered variants was obtained as previous described [28].

To assess the sensitivity of the method dilution curves were generated starting from heterozygous reference standards by Diatech Pharmacogenetics (50% mutated allele) mixed with wild-type DNA in a proportion mimicking the concentration of tumor DNA in plasma of cancer patients.

Finally, a blind analysis on 18 formalin-fixed paraffin-embedded (FFPE) samples previously genotyped [28] and 4 plasma samples were performed with this procedure.

All the plasma samples were previously analyzed by biopsy on tumor tissue by the Departmentof of Pathology at the San Raffaele Hospital by MassARRAY (Sequenom, San Diego, CA, USA), that is reported to have a sensitivity of 5% in the detection of KRAS mutations. The study was approved by the Institutional Review Board of the San Raffaele Hospital in Milan and all clinical investigation has been conducted according to the principles expressed in the Declaration of Helsinki. Informed written consent has been obtained from the participants.

### PCR conditions

The DNA sequences containing the mutations were amplified using 5’-biotin forward and 5’-tagged reverse primers.

Primer sequences, amplification length and annealing temperature (Ta) for the *KRAS* (codon 12–13, 61 and 146), *NRAS* (codon 12–13) and *BRAF* (codon 600) were detailed in S1 Table.

PCR was performed in 25 μL reaction containing 15 ng of tissue DNA, 200 μM of each deoxynucleotide, 10 mM Tris–HCl (pH 8,3), 50 mM KCl, 1.5 mM MgCl_2_, 1 U of DNA polymerase (FastStart Taq, Roche) and 10 pmoles of each primer. Cycling conditions entailed an initial denaturation at 95°C for 4 min followed by 35 cycles at 95°C for 30 s, 58°C for 30 s and 72°C for 30 s, and a final elongation at 72°C for 10 min.

Concerning the plasma samples, the PCR was performed in 50 μL of reactions containing 15 μl of DNA extracted from plasma, 200 μM deoxynucleotide triphosphates, 10 mM Tris–HCl (pH 8.3), 50 mM KCl, 1.5 mM MgCl_2_, 1.3 U of DNA polymerase (FastStart Taq, Roche) and 20 pmoles of each primer.

Cycling conditions were as follows: 95°C for 4 min; 47 cycles at 95°C for 30 s, 58°C for 30 s, 72°C for 30 s and 72°C for 10 min. Each sample was amplified in triplicate.

### Silicon slide coating and microarray preparation

Untreated silicon 1000Å Thermal Oxide chips (14 X 14 mm) and slides (75 X 25 mm) were supplied by SVM, Silicon Valley Microelectronics Inc. (Santa Clara, CA USA). After an activation treatment (15 min) with oxygen plasma, the silicon chips or slides were immersed for 30 min in solution (1% w/v in 0.9 M (NH4)_2_SO_4_ water solution) of a modified form of copoly (DMA-NAS-MAPS) with 10% NAS moiety. The copoly (DMA-NAS-MAPS) was synthesized and characterized as previously described [29] but to enhance the binding capacity of the copoly (DMA-NAS-MAPS) the N-acryloyloxysucinimide (NAS) molar fraction was increased from 2% to 10%. The slides were finally rinsed with water and dried under vacuum at 80°C for 20 minutes. We selected twenty-seven different oligonucleotide sequences from the GeneFlex Tag Array collection (Affymetrix, Santa Clara, CA) that contains sequence information for 2000 oligonucleotides with minimal tendency for cross-hybridization, as capture probes spotted on silicon chips corresponding to thirteen *KRAS* mutations, eight *NRAS* mutations and one *BRAF* mutation and to the wild-type sequences (See S2 Table). The capture probes and a control oligonucleotide (COCU8), used as reference spots, amino modified at the 5’ end, from Metabion International AG (Steinkirchen, Germany), were dissolved in the printing buffer (150 mM sodium phosphate pH 8.5, 0.01% Sucrose monolaurate) at a concentration of 10 μM and printed by a piezoelectric spotter, SciFLEX ARRAYER S5 (Scienion, Berlin, Germany). Spotting was performed at 20° C in an atmosphere of 60% humidity. After the spotting step the chips were incubated overnight and all residual reactive groups of the coating polymer were blocked as described in [30].

### Preparation of single-strand DNA from PCR products with streptavidin magnetic beads and liquid allele-specific hybridization

Prior to use, the streptavidin-coated magnetic beads (Dynabeads M-270 Streptavidin, Invitrogen) were washed three times with Binding and Washing buffer (B&W) (5 mM Tris-HCl, pH 7,5; 0,5 mM EDTA; 1 M NaCl) according to the manufacturer’s protocol. Afterwards the streptavidin-coated beads (250 μg) were added to a PCR tube containing 25 μL of biotinylated PCR product and 75 μL of B&W buffer and incubated at room temperature for 10 minutes with gentle rotation. The beads with the bound PCR products were then washed 2–3 times in the B&W buffer and resuspend in 40 μL of the same buffer and heated to 95°C for 5 minutes. At the end of the denaturation step, the PCR tube was placed on a magnet and the supernatant with the single-strand PCR (~ 36 μL) was transferred to a fresh tube containing 1 μL of a10 μM stabilizer, an oligonucleotide necessary to open the secondary structures present in the amplicon (final concentration 0,3 μM) and incubated with this oligonucleotide for 10 minutes at room temperature, while the beads with the bound biotinilated PCR strand were discharged. Then, for the detection of wild-type and *KRAS*, *NRAS* and *BRAF* mutations, the reporters for the wild-type and the mutated sequences were added together in equimolar amounts (final concentration 0,1 μM) to the tube containing the ssPCR-stabilizer solution (final volume 40 μL). The incubation lasted for 35 minutes with gentle rotation in a stepwise gradient of temperature ranging from 42°C to 29°C. The sequences of the spotting probes, the reporters, the stabilizers and Universal-Cy3 are reported in S2 Table.

### Microarray hybridization, image scanning and data analysis

After the liquid allele-specific hybridization to detect the mutations, the universal oligonucleotide labelled with Cy3 (Universal-Cy3) and a Cy3-labeled oligonucleotide (COCU10) complementary to COCU8, were added to the ssPCR-reporters solution to a concentration of 0,3 μM and 0,01 μM respectively. The solution was then spread onto the spotted silicon chips and cover slips were placed on the spotted area. The chips were incubated at room temperature for 15 min in a humid hybridization chamber.

Finally, the silicon chips were removed from the hybridization chamber, washed and scanned as reported in [28]. Data intensities were extracted with the scanner (Scanarray Express) and the data analysis was performed for each experiment.

## Results and discussion

### Overview of the method

The classical microarray genotyping approach, based on specific capture sequences immobilized on the surface of a glass slide, fails in detecting minority point mutations. When a small amount of mutated sequence is present in a large amount of wild-type background, high analytical sensitivity and specificity are required. In classical SNP microarray detection, after denaturation, only one PCR strand is captured on the surface. However, due to re-annealing with the complementary strand and steric hindrance of the surface, the capture efficiency is extremely low with dramatic consequences on assay sensitivity. Multiplexing the mutations detected is even more challenging, as it is difficult to design capture reporters that bind selectively to their complementary PCR strand at a single temperature. In this study, we overcome both problems with a sequence of operations that render the assay highly sensitive, multiplexable and robust.

**Fig 1:**
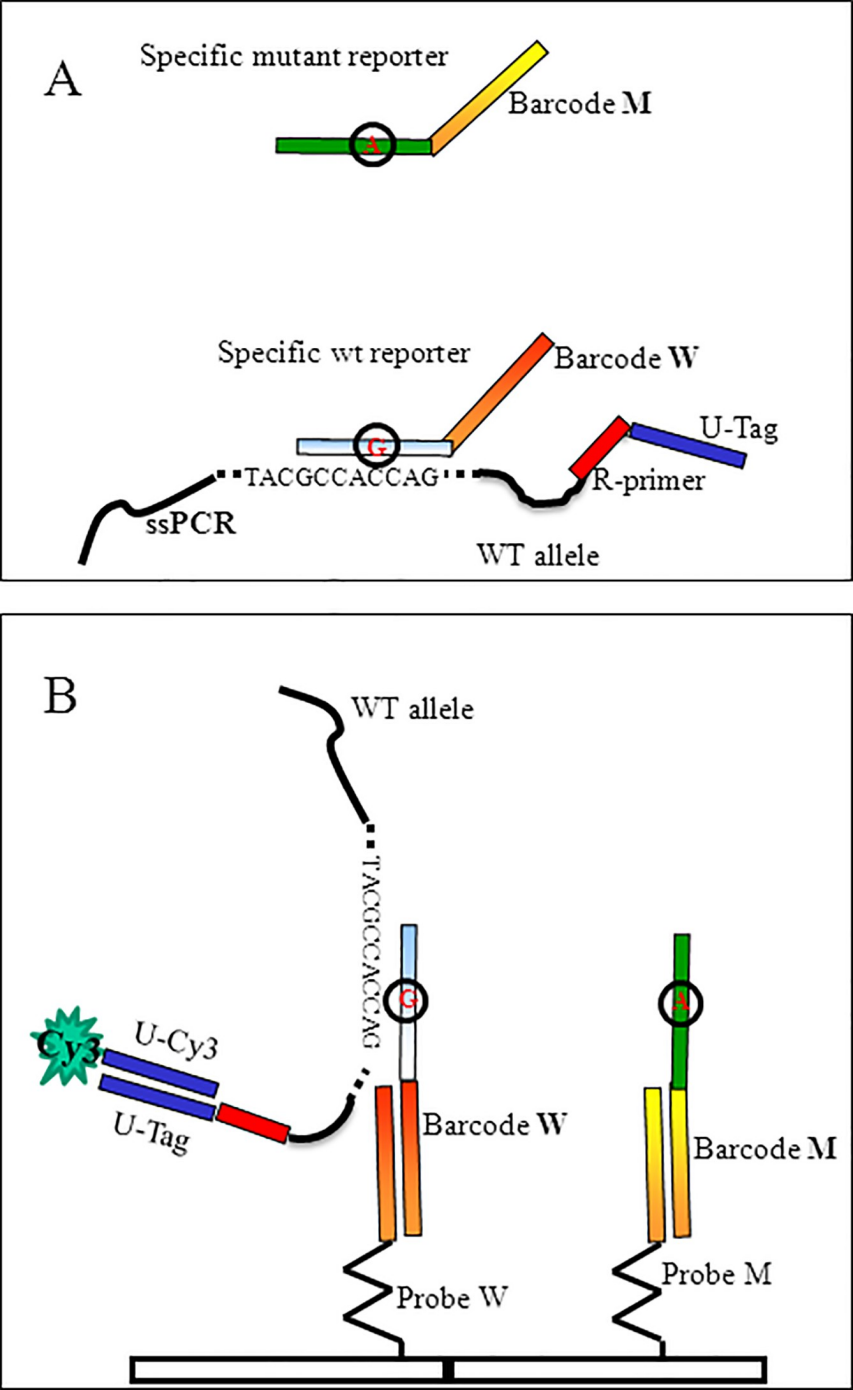
Scheme of the assay. (A) In the case of *KRAS* wild-type allele, the specific wild-type reporter hybridizes in solution with the single strand PCR product (ssPCR), whereas the specific mutant reporter (with the variant position circled and in red) does not. (B) Different barcodes in the 3’ domain of the reporter sequences (Barcode W for wild-type, Barcode M for mutation) direct the ssPCRs to different position on the array. The position in the array is revealed when the U-tag sequence at 5’-end of the ssPCR interacts with the complementary Cy3-labeled oligonucleotide, Universal-Cy3 (U-Cy3), added in the last step of the assay.

The scheme of the assay is shown in Fig 1. After amplification, the biotinylated double stranded PCR product was bound to streptavidin-coated magnetic beads and captured on a magnet. Following thermal denaturation, the single strand PCR product was released in the supernatant and recovered while the DNA strand bound to the beads was captured with a magnet and discarded. The ssDNA was hybridized in solution with a stabilizer oligonucleotide to open and stabilize the secondary structures eventually present on the DNA strand and then with specific oligonucleotide reporters (see S2 Table) whose sequence consists of two domains. The 5’ domain corresponds to a segment of *KRAS*, *NRAS* or *BRAF* sequence (wild-type or mutant reporters) whereas the 3’ domain is a synthetic “barcode” sequence that recognizes complementary oligonucleotide probes arrayed at specific locations on the silicon chip (barcode probe). Following hybridization, the tagged PCR was captured on the surface of the array through its specific barcode domain and revealed with a universal Cy3-oligonucleotide complementary to the tag present at the 5’-end of the single stranded PCR (U-tag).

The key to achieve successful genotyping by this technique was to decouple mutation sequence recognition from surface capture. To obtain a correct genotyping without false positives/negatives, in standard microarray assays, the surface hybridization must be carried out at a specific temperature. For example, in a previous work we reported seven different temperatures for the detection of *KRAS* codon 12 and 13 mutations [28]. The sequence dependence of the probe/target melting temperature limits the number of mutations that can be detected simultaneously. Even though a careful optimization of probe length and sequence can partially overcome this problem, it is difficult to find optimal conditions for all mutations. In our new approach, the hybridization with mutation specific sequences is carried out in solution in a thermal gradient ranging from 42°C to 29°C. In this gradient each reporter oligonucleotide binds to the amplicon in optimal conditions. When the single strand PCR bound to the dual-domain reporters are captured onto the microarray surface, less stringent conditions are required and the same temperature applies to all the barcodes. Another important feature of this system is the surface chemistry used to bind the 20mer oligonucleotide barcodes to the surface as well as the use of a silicon/silicon oxide substrate that enhances fluorescence [31]. A three-dimensional coating made of copoly (DMA-NAS-MAPS), a copolymer well known for its high binding capacity and low non-specific adsorption, was used [29]. Also the selection of the barcode probe sequences is important because it affects the efficiency and the specificity of the detection.

### Optimization of genotyping

**Fig 2:**
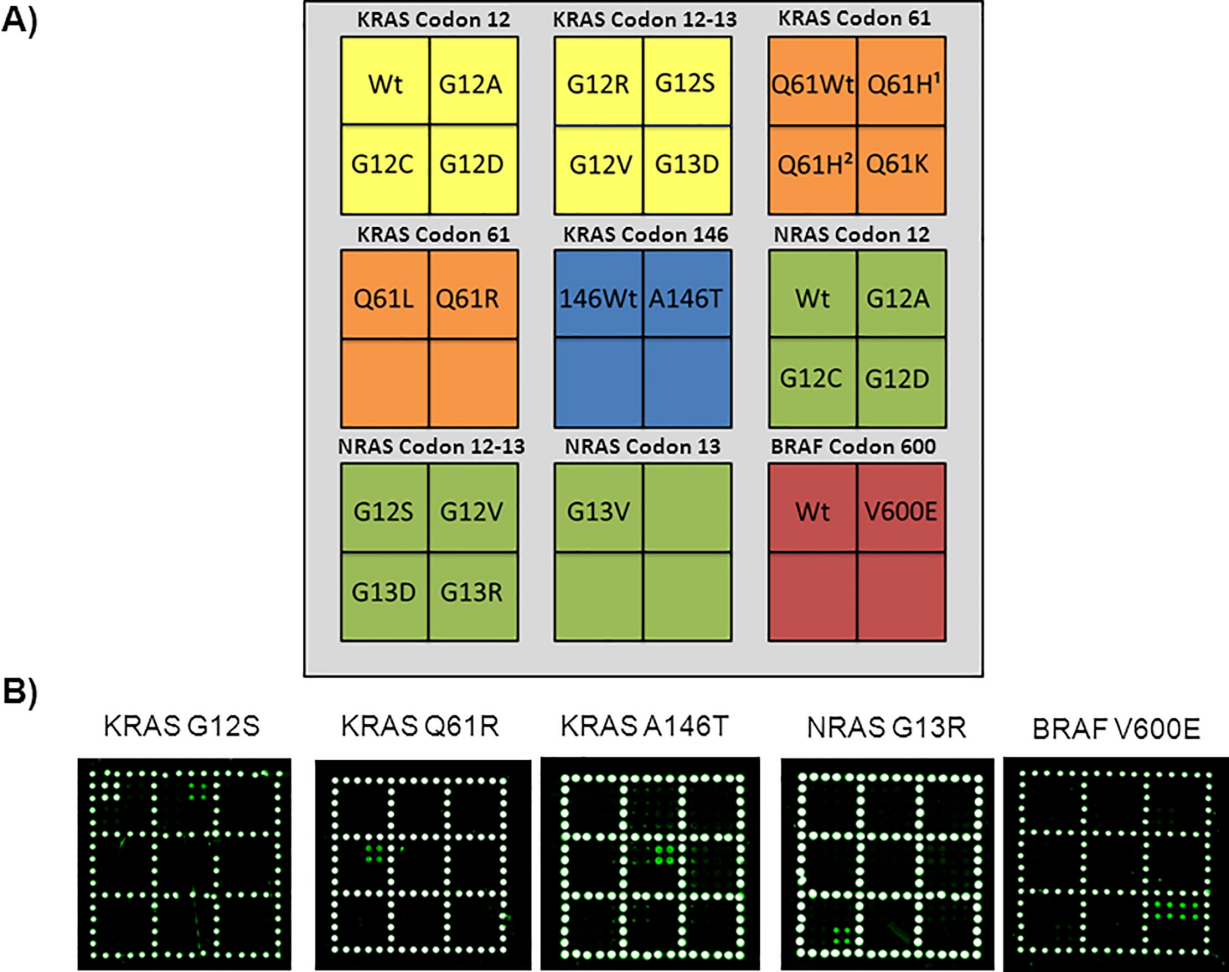
Examples of genotyping of *KRAS*, *NRAS* and *BRAF* mutations. A) Schematic representation of the spotted barcode probe array. Silicon chips coated with copoly(DMA-NAS-MAPS) are used as substrates for the covalent attachment of amino-modified barcode probe oligonucleotides arrayed at discrete locations. Each position in the grid identifies an individual barcode probe address corresponding to *KRAS* codon 12–13, *KRAS* codon 61 (Q61H^1^ c.183A>C, Q61H^2^ c.183A>T), *KRAS* codon 146, *NRAS* codon 12–13 and *BRAF* mutations. The light grey portion of the array is spotted with an amino-modified oligonucleotide (COCU8), not correlated with the genes, to be used as reference spots. B) Microarray scanning of the Cy3 fluorescence signal of five different silicon chips. Each robotically spotted array is hybridized with an individual single strand PCR incubated with the whole set of dual-domain reporters. The fluorescence detection is obtained incubating the array with a mixture of the universal Cy3 labeled oligonucleotide complementary to the tagged-reverse primer of the single strand PCR and with a Cy3-labeled oligonucleotide (COCU10) complementary to COCU8. *KRAS* G12S, *KRAS* Q61R, *KRAS* A146T, *NRAS* G13R and *BRAF* V600E correspond to the control sample containing the indicated mutation. All the five samples of known genotype (mutant homozygous or heterozygous in case of *KRAS* G12S and *BRAF* V600E) were correctly identified.

We developed a microarray covering the 22 mutations as well as wild-type controls listed in the Sample section above. Fig 2 shows the spotting scheme of the array and typical examples of genotyping of *KRAS* (G12S, Q61R and A146T), *NRAS* (G13R) and *BRAF* (V600E) mutations from reference or mutagenized controls. We succeeded in correctly genotyping all the *KRAS*, *NRAS* and *BRAF* mutations considered. As shown in Fig 2, an intense fluorescence signal appears only at the location where the immobilized oligonucleotide is complementary to the barcode domain of the reporter sequence that captures the PCR with very low cross-hybridization and a good reproducibility from spot to spot. The assay was optimized also by analysing DNA extracted from Formalin-Fixed Paraffin-Embedded (FFPE) clinical samples previously characterized by the QX100 Droplet Digital PCR (ddPCR) System (Bio-Rad) [28]. FFPE samples from subjects either mutated in *KRAS* codon 12 (G12A, G12C, G12D, G12R, G12S, G12V), 13 (G13D), 61 (Q61L) and NRAS codon 12 (G12D) or wild-type were unambiguously genotyped as shown in S1 and S2 Figs.

### Detection limit

**Fig 3:**
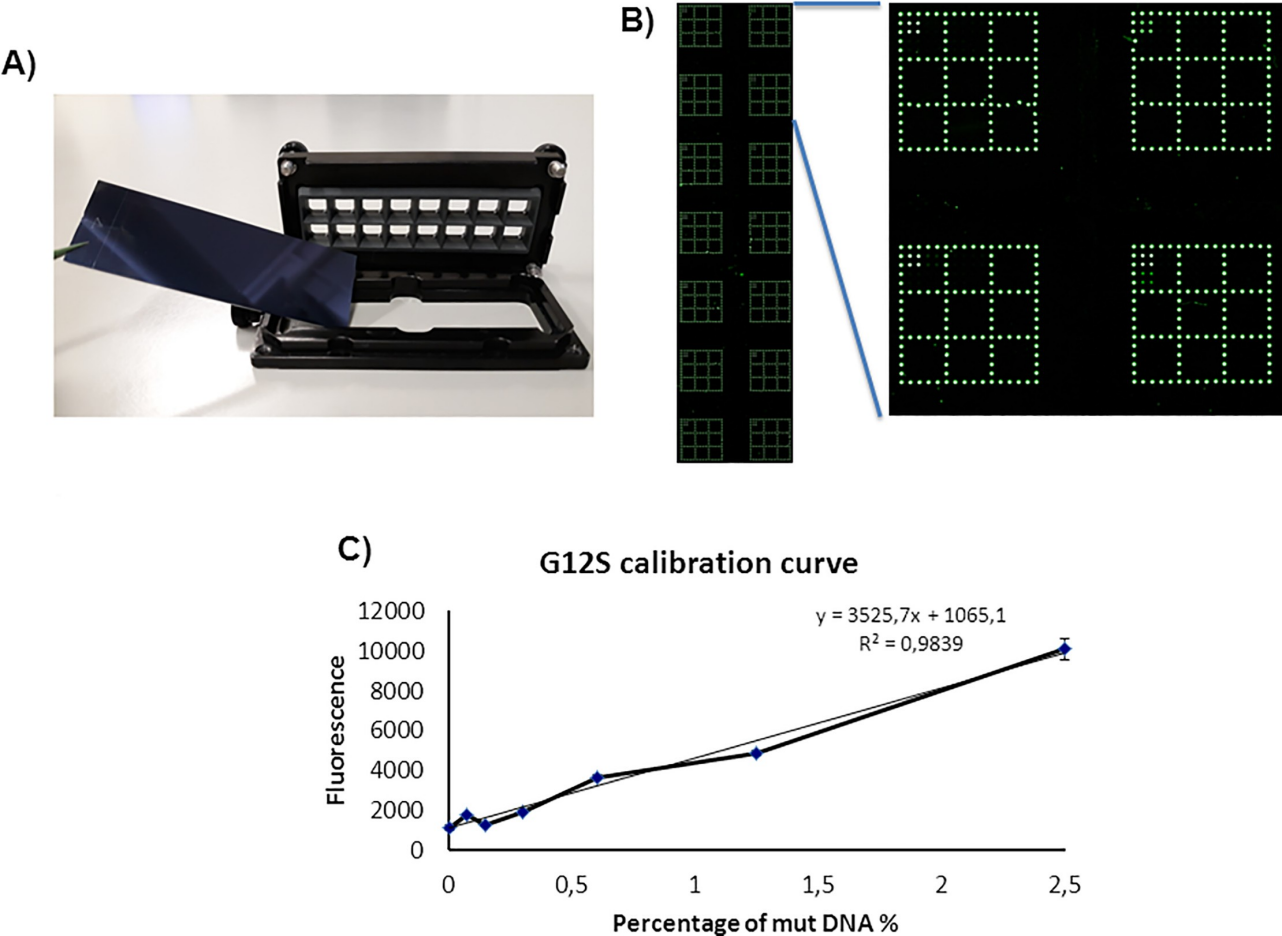
G12S calibration curve. A) Picture showing the setup utilized to realize the calibration curves for the *KRAS* codon 12–13 mutations. B) Microarray scanning of the Cy3 signal of the coated silicon slide and the magnification of a portion of it showing the result of the hybridization of four different concentration of mutant DNA in four different wells. C) Plot representing the relative fluorescence intensity of the signal corresponding to the Cy3-labeled mutated single strand PCR bound to the G12S barcode probe. The points, calculated as the average of the intensity of four spots, correspond to the percentage of the *KRAS* G12S mutation in a background of KRAS wild-type DNA. The value at point 0 represents the relative fluorescence intensity of the background presents on the G12S barcode probe array of the well hybridized with wild-type control sample. The error bars are the standard deviations of the fluorescence intensity of each well. The equation of the trend line of the graph is utilized to extrapolate the limit of detection (LOD) for the assay.

The PCR sequencing method, which is generally considered a gold standard for clinical diagnosis, is reliable only when the percentage of mutant-to-wild type reaches 10%-20% [32]. In tissue biopsy it is very difficult to obtain homogenous tumor samples and sometime the mutation content can be below the limit of detection of PCR sequencing. Even more challenging is the analysis of ctDNA because of its low mutant allele frequency and large dynamic range. The level of ctDNA in cancer patients ranges from <0.1% to >50% out of the total cfDNA. Therefore, the technical sensitivity and dynamic range of the assay are critical [33]. Thus, more sensitive mutation-testing methods are urgently needed to improve clinical diagnosis. We evaluated the sensitivity of our method with serial dilutions (2,5%; 1,25%; 0,62%; 0,31%; 0,15% and 0,075%) of mutated DNA opportunely mixed with wild-type DNA. We prepared the mixtures starting from the heterozygous reference standards by Diatech Pharmacogenetics (50% mutated G12A, G12C, G12D, G12R, G12S, G12V and G13D alleles) with a constant input of 20 ng of total DNA for all the mutations. To build the calibration curves for each of the seven most common mutations of the *KRAS* gene, we spotted the barcode probes shown in Fig 2A in 14 replicates on a coated silicon slide (one slide for each mutations) and then we used to separate the dilutions a commercial incubation chamber (Nexterion IC-16, SCHOTT) (Fig 3A). Samples with increasing percentage of mutated DNA were hybridized simultaneously in different wells (Fig 3B). The value of fluorescence intensity detected for each of the seven *KRAS* codon 12–13 mutations together with the background fluorescence of the control sample (wild-type DNA) were plotted versus the percentage of mutated DNA. Fig 3C shows an example of calibration curve for the KRAS G12S mutation. The LODs (lowest percentage of detectable mutated DNA) extrapolated for each mutation are reported in Table 1. The determination of LOD is based on the equation: 3,3*σ*/*s* where *s* is the slope of calibration curve and *σ* is the standard deviation of fluorescence background in wild-type sample. The LODs found with this system range from 0,03% for *KRAS* G12C and G12D mutations to 0,28% for *KRAS* G13D mutation. This is a significant improvement over the sensitivity of PCR sequencing which could detect mutations only when present at 10%. The high sensitivity achieved makes it possible to apply the method to single mutation detection in ctDNA. We choose to demonstrate the sensitivity of our method in *KRAS* codon 12–13 mutations because they are highly abundant in mCRC being present in 35%-56% of cases whereas the frequency of *NRAS* mutations is between 1%-7%. Moreover, the majority of *KRAS* mutations are in codon 12 (80%) and 13 (15%) of exon 2; mutations in other positions of the *KRAS* gene, such as codon 61 and 146, are much less frequent, representing only ~ 1% [34].

**Table 1:** The extrapolated limits of detection for the seven most common mutations of the KRAS gene.

KRAS mutation	LOD %
**G12A**	0,07
**G12C**	0,03
**G12D**	0,03
**G12R**	0,07
**G12S**	0,13
**G12V**	0,09
**G13D**	0,28

### Detection of *KRAS* mutations in Formalin-Fixed Paraffin-Embedded (FFPE) cancer tissues and in cell-free circulating tumor DNA (ctDNA)

**Fig 4:**
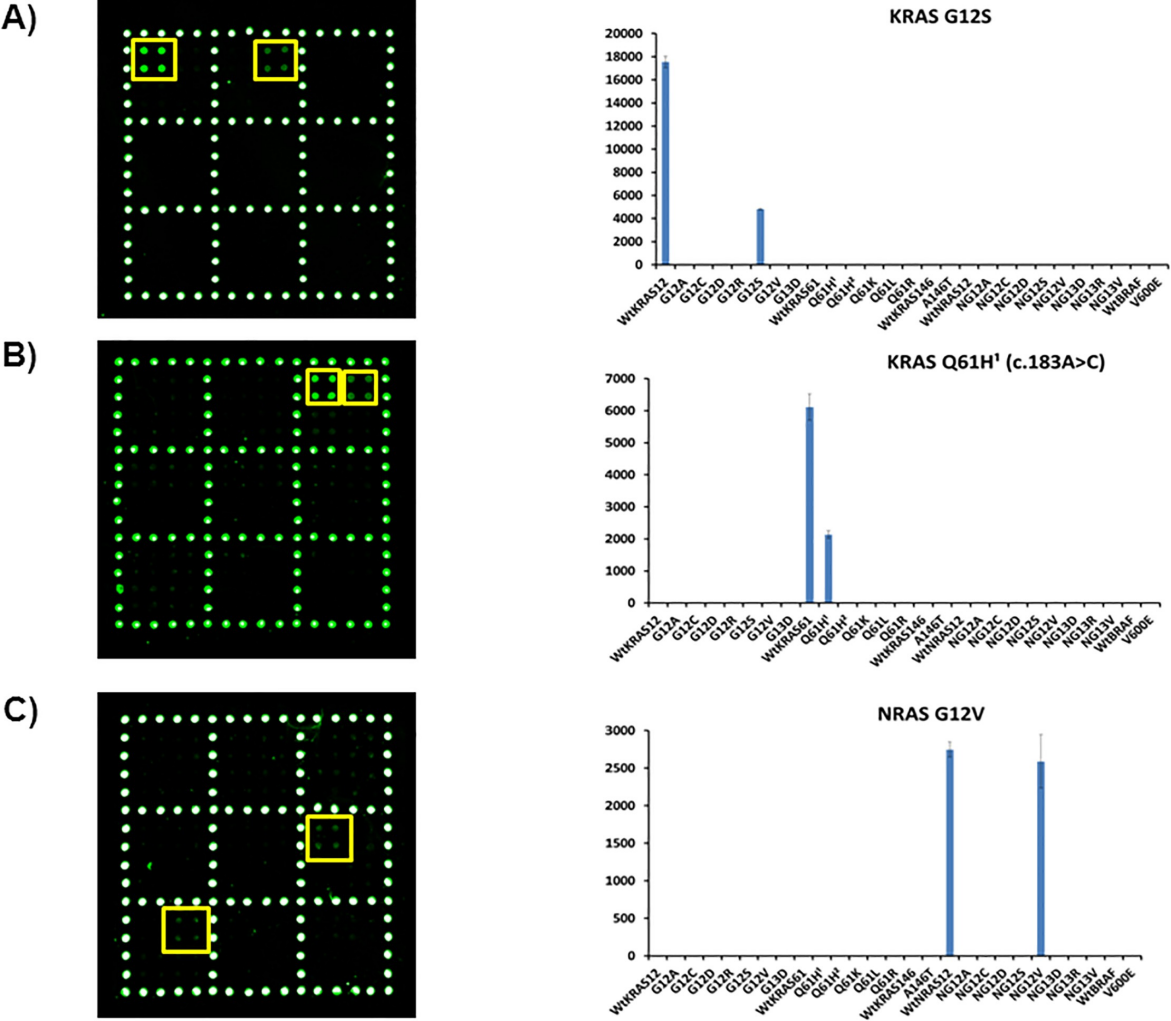
Analysis of DNA extracted from Formalin-Fixed Paraffin-Embedded (FFPE) clinical samples. The spotting schema of the barcode sequences is the same of Fig 2A. (A) Cy3 fluorescence image and the plot of the relative fluorescence intensity of the sample identified with *KRAS* G12S mutation. (B) Cy3 fluorescence image and the plot of the relative fluorescence intensity of the sample identified with *KRAS* Q61H^1^ (c.183A>C) mutation. (C) Cy3 fluorescence image and the plot of the relative fluorescence intensity of the sample identified with *NRAS* G12V mutation. The yellow squares in the images were used to highlight more easily the analyzed spots. The bars are the average of the intensity of the 4 spots (2 X 2 subarray) of each barcode probe subarrays. The error bars are the standard deviations of the fluorescence intensity of each sample. Q61H^1^ c.183A>C, Q61H^2^ c.183A>T.

The assay was validated by blind analysis of DNA extracted from 18 Formalin-Fixed Paraffin-Embedded (FFPE) clinical samples and liquid biopsy samples. In order to correctly genotype the tumour DNA with a single chip, we hybridized single stranded PCR of the different samples with a mixture of all specific reporters (22 mutant reporters and 5 wt reporters) in a stepwise gradient of temperature ranging from 42°C to 29°C. The mixture was kept for 5 minutes at 42, 37, 36, 30 and 29°C. All the 18 samples were analysed in parallel and correctly genotyped in less than 90 minutes. Typical results are shown in Fig 4 for the samples identified with *KRAS* G12S mutation (A), *KRAS* Q61H (c.183A>C) mutation (B) and NRAS G12V mutation (C). The corresponding wild-type is always present while different 2X2 spot replicates are present at different locations on the slide depending on the mutation detected.

**Fig 5:**
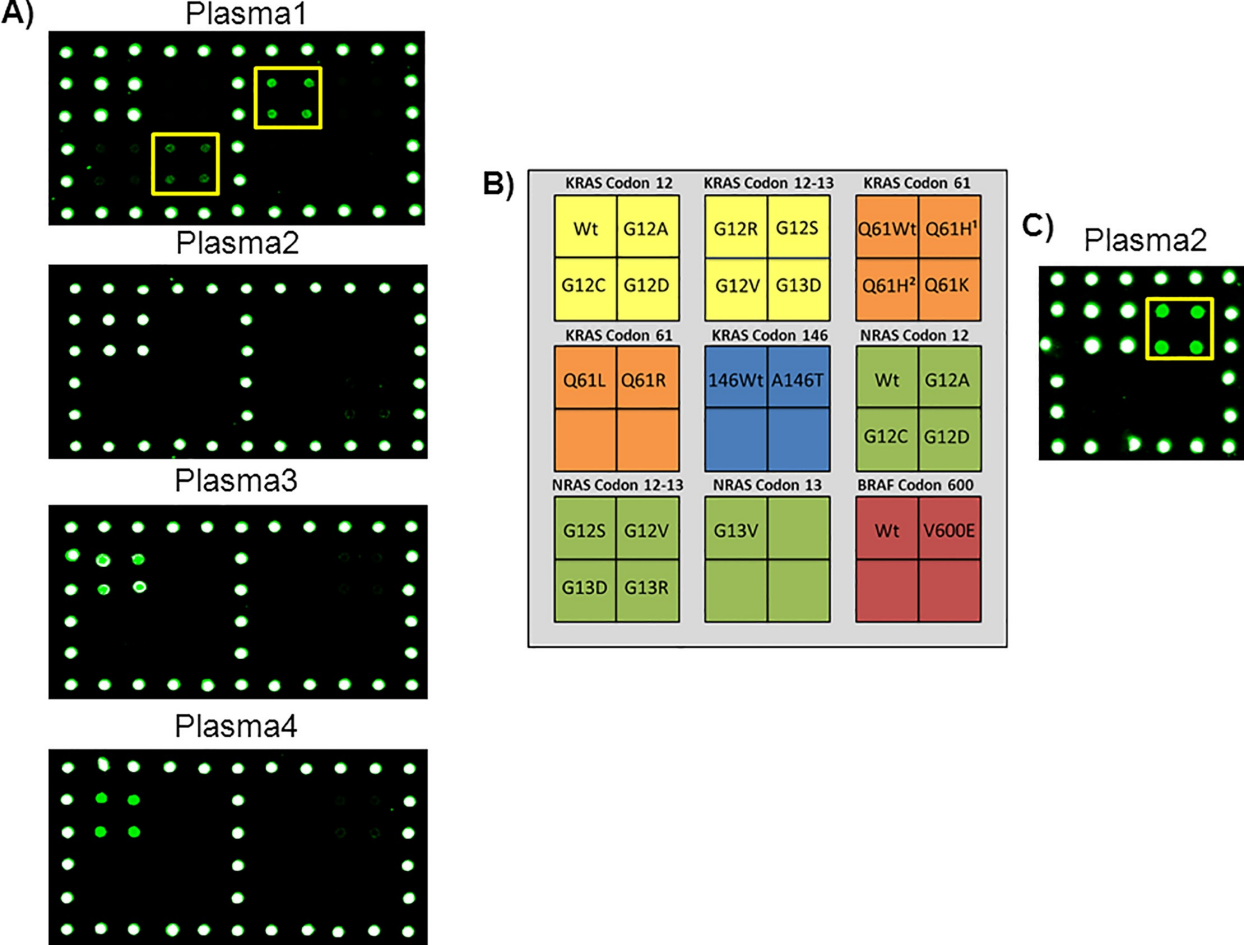
Analysis of ctDNA extracted from plasma of 4 patients with metastatic colorectal cancer. A) Microarray scanning of the Cy3 fluorescence signal of four different plasma samples. Only the part of the array corresponding to the barcode probes for *KRAS* codon 12 and 13 is shown. In Plasma1 the barcode probes corresponding to *KRAS* G12D and G12R mutation are highlighted. B) Schematic representation of the spotted barcode probe array. Q61H^1^ c.183A>C, Q61H^2^ c.183A>T C) Microarray scanning of the Cy3 fluorescence signal of the *BRAF* codon 600 area corresponding to the *BRAF* barcode probes for the Plasma 2 sample. The frame highlights the barcode probes corresponding to the *BRAF* V600E mutation.

Finally, we performed a proof of concept analysis of ctDNA extracted from plasma of 4 patients with metastatic colorectal cancer to demonstrate the feasibility of our methodology as a tool for liquid biopsy. The results of the analysis are shown in Fig 5. After having been analyzed with our approach the same samples were assayed also by ddPCR. Interestingly, the plasma number 1, which according to the Pathological Anatomy was mutated in G12D, was found to be mutated correctly in G12D but also in G12R. Digital PCR confirmed the presence of G12R mutation with a fractional abundance of 0,95%. Interestingly this mutation was not detected in the tissue biopsy. The fractional abundance of the G12D mutation found by ddPCR was 0,036%, a value close to the limit of detection of our method but still visible. The discordance between tissue and liquid biopsy may have a biological significance. Moreover, considering the heterogeneity of solid tumors, we cannot exclude that the tumor section that was selected for tissue biopsy analysis may not have been representative of the total tumor population. In the other 4 plasma samples there was perfect concordance between tissue biopsy, our method and ddPCR: plasma 2 was wild-type in *KRAS* but mutated in *BRAF* codon V600E, while plasma 3 and 4 were wild-type for all the genes analyzed.

## Conclusions

In summary, we describe a microarray platform for rapid, specific and sensitive detection of the most common mutations in the *KRAS*, *NRAS* and *BRAF* genes suitable for high-throughput analysis without costly instrumentation.

To the best of our knowledge, this is the first time that a microarray based analysis reaches the level of sensitivity reported by this work in the detection of minority mutations in a multiplex assay able to genotype a large number of mutations in a single test both in tissue biopsy samples and in circulating tumor DNA from patients with mCRC. The most significant advantage of our system is the ability to separate the mutation detection from the array hybridization without the use of enzymatic reactions such as ligases or single base extensions. Direct hybridization DNA microarrays suffer from poor selectivity due to mismatch hybridization and non-specific binding. In contrast, our approach, can readily distinguish point mutations in solution, thanks to the temperature gradient, and then the use of divergent surface barcode probe sequences with similar properties allows rapid hybridizations of the array at room temperature. Using the tag-microarray method, the genotyping of clinical sample, can be obtained in less than 90 minutes, a time significantly shorter than that of direct sequencing, which is generally considered a gold standard for clinical diagnosis, which normally takes 1–2 working days.

Finally, it is worth noticing the versatility of this approach that could be defined "universal": several mutations can be detected using the same barcoded microarray simply by changing the portion of the dual domain reporter that is complementary to the mutated sequence. Thus, this innovative technique is suitable for routine diagnosis of a wide range of genetic variations.

## Supporting information

S1 Fig Genotyping of control Formalin-Fixed Paraffin-Embedded (FFPE) clinical samples. The plots of the relative fluorescence intensity after hybridization of six control clinical samples with six spotted chips are represented. *KRAS* G12A, C, D, R, S, and V indicate the Formalin-Fixed Paraffin-Embedded (FFEP) genotype. Q61H^1^ c.183A>C, Q61H^2^ c.183A>T. All the bars are the average of the intensity of the 4 spots (2 X 2 subarrays) of each barcode probe subarrays. The error bars are the standard deviations of the fluorescence intensity of each subarray.
(TIF)

S2 Fig Genotyping of control Formalin-Fixed Paraffin-Embedded (FFPE) clinical samples. The plots of the relative fluorescence intensity after hybridization of three control clinical samples with three spotted chips are represented. *KRAS* G13D, Q61L, and *NRAS* G12D indicate the Formalin-Fixed Paraffin-Embedded (FFEP) genotype. Q61H^1^ c.183A>C, Q61H^2^ c.183A>T. All the bars are the average of the intensity of the 4 spots (2 X 2 subarrays) of each barcode probe subarrays. The error bars are the standard deviations of the fluorescence intensity of each subarray.
(TIF)

S1 Table Primer sequences, amplification length and annealing temperature (Ta) for the *KRAS* (codon 12–13, 61 and 146), *NRAS* (codon 12–13) and *BRAF* (codon 600). (DOCX)

S2 Table Sequences of spotted probes and reporters. (DOCX)
