# Supplementary figures and images for: Analysis of KRAS, NRAS and BRAF mutational profile by combination of in-tube hybridization and universal tag-microarray in tumor tissue and plasma of colorectal cancer patients
Source: PLoS One. 2018 Dec 18;13(12):e0207876. doi: 10.1371/journal.pone.0207876 (PMC6298683; doi:10.1371/journal.pone.0207876)

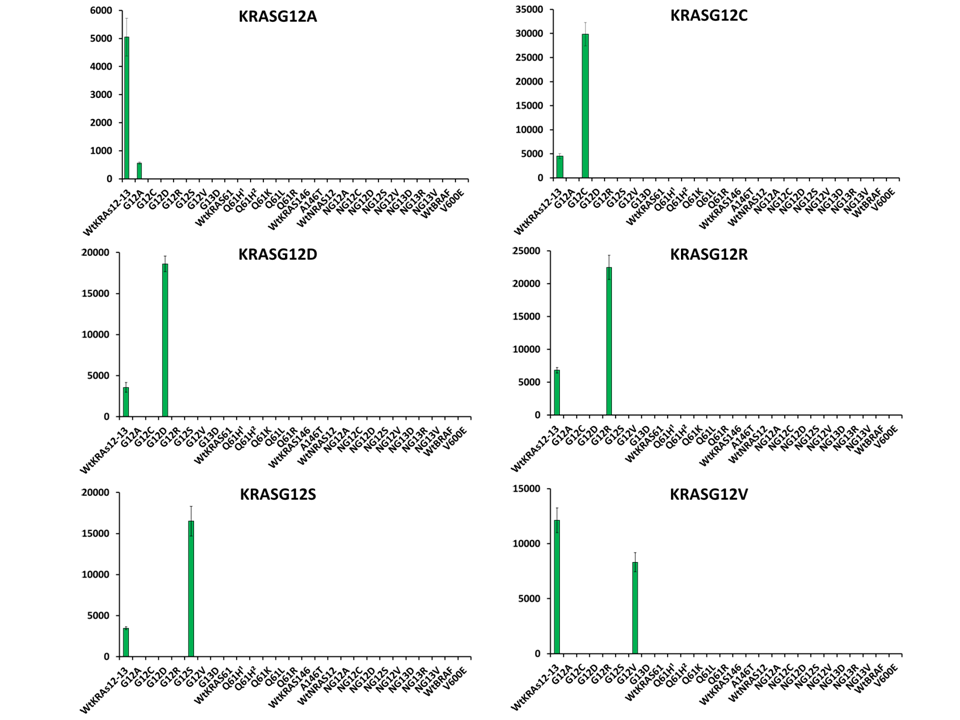

Supplement: S1 Fig — The plots of the relative fluorescence intensity after hybridization of six control clinical samples with six spotted chips are represented. KRAS G12A, C, D, R, S, and V indicate the Formalin-Fixed Paraffin-Embedded (FFEP) genotype. Q61H1 c.183A>C, Q61H2 c.183A>T. All the bars are the average of the intensity of the 4 spots (2 X 2 subarrays) of each barcode probe subarrays. The error bars are the standard deviations of the fluorescence intensity of each subarray. (TIF) [file pone.0207876.s001.tif]

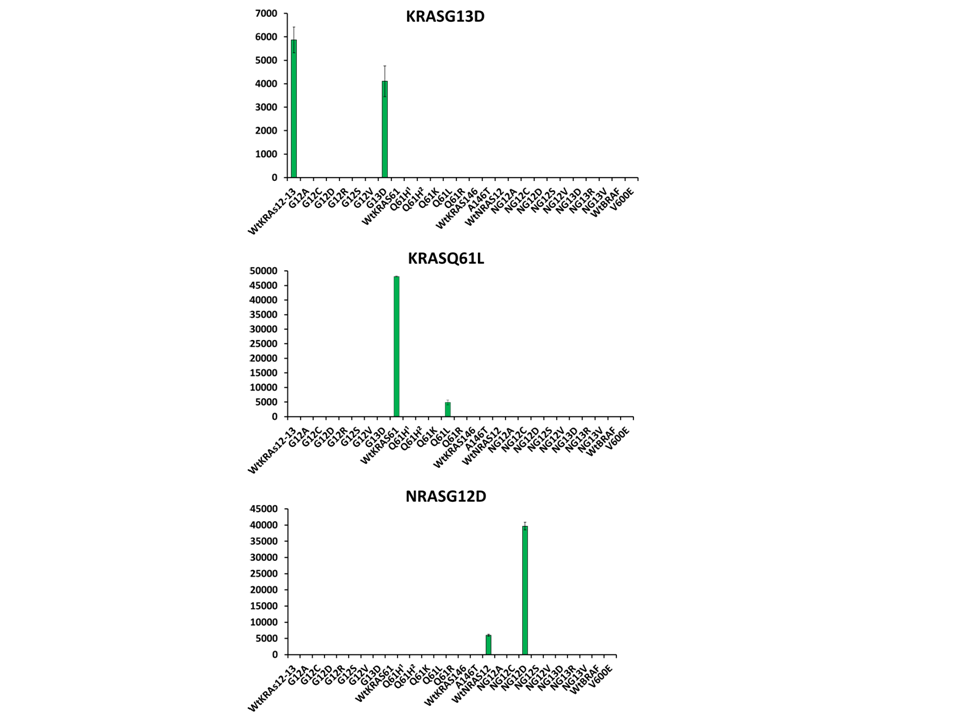

Supplement: S2 Fig — The plots of the relative fluorescence intensity after hybridization of three control clinical samples with three spotted chips are represented. KRAS G13D, Q61L, and NRAS G12D indicate the Formalin-Fixed Paraffin-Embedded (FFEP) genotype. Q61H1 c.183A>C, Q61H2 c.183A>T. All the bars are the average of the intensity of the 4 spots (2 X 2 subarrays) of each barcode probe subarrays. The error bars are the standard deviations of the fluorescence intensity of each subarray. (TIF) [file pone.0207876.s002.tif]
