# Supplementary material for: Analysis of KRAS, NRAS and BRAF mutational profile by combination of in-tube hybridization and universal tag-microarray in tumor tissue and plasma of colorectal cancer patients
Source: PLoS One. 2018 Dec 18;13(12):e0207876. doi: 10.1371/journal.pone.0207876 (PMC6298683; doi:10.1371/journal.pone.0207876)
Supplement: S1 Table — (DOCX) [file pone.0207876.s003.docx]

**Supporting Information**

**Supplemental Data Table 1. Sequences of primers**

| **GENE**  codon | **Primers sequence** | **Fragment lenght**  **(bp)** | **Ta**  **(°C)** |
| --- | --- | --- | --- |
| ***KRAS***  codon 12-13 | For 5’-GCCTGCTGAAAATGACTGAA-3’  Rev 5’-*AGAATGGTCCTGCACCAGTAA-3’ | 167 | 58 |
| ***KRAS***  codon 61 | For 5’-ATTCCTACAGGAAGCAAGTAG - 3’  Rev 5’-*GCCCTCCCCAGTCCTCA - 3’ | 119 | 58 |
| ***KRAS***  codon 146 | For 5’- TAAGGACTCTGAAGATGTAC -3’  Rev 5’-*TAAATGACATAACAGTTATGAT-3’ | 205 | 58 |
| ***NRAS***  codon 12-13 | For 5’-GTTCTTGCTGGTGTGAAATGACTG- 3’  Rev 5’-*CCTCACCTCTATGGTGGGATCATAT- 3’ | 134 | 58 |
| ***BRAF***  codon 600 | For 5’- TGCTTGCTCTGATAGGAAAATG -3’  Rev 5’-*CCA CAA AAT GGA TCC AGA CA-3’ | 173 | 58 |

*Each reverse primer is tagged in 5’ with the following sequence: CTGAGTCCGAACATTGAG
