# Supplementary material for: Analysis of KRAS, NRAS and BRAF mutational profile by combination of in-tube hybridization and universal tag-microarray in tumor tissue and plasma of colorectal cancer patients
Source: PLoS One. 2018 Dec 18;13(12):e0207876. doi: 10.1371/journal.pone.0207876 (PMC6298683; doi:10.1371/journal.pone.0207876)
Supplement: S2 Table — (DOCX) [file pone.0207876.s004.docx]

**Supplemental Data Table 2. Sequences of spotted probes and reporters**

| **KRAS Mutations** | | | |
| --- | --- | --- | --- |
| **Exon 2 Codon12-13**  **(Amino acid change)** | **Spotted capture probes^1^** | **Reporter sequences** | |
| Wild-type | 5’-actccagtgccaagtacgat-3’ | 5’-^*^CTGGTGGCGTA-^†^atcgtacttggcactggagt-3’ | |
| c.35G>C (p.G12A) | 5’-cgatccgattacaggccgat-3’ | 5’-^*^TGGAGCTG**C**TG-^†^atcggcctgtaatcggatcg-3’ | |
| c.34G>T (p.G12C) | 5’-taatcttaattctggtcgcgg-3’ | 5’-^*^ CT**T**GTGGCGTAG-^†^ccgcgaccagaattagatta-3’ | |
| c.35G>A (p.G12D) | 5’-ggctcacgtcttatttgggc-3’ | 5’-^*^GCTG**A**TGGCGT-^†^gcccaaataagacgtgagcc-3’ | |
| c.34G>C (p.G12R) | 5’-tcttctagttgtcgagcagg-3’ | 5’- ^*^CT**C**GTGGCGTA-^†^cctgctcgacaactagaaga-3’ | |
| c.34G>A (p.G12S) | 5’-attgaccaaactgcggtgcg-3’ | 5’-^*^GCT**A**GTGGCGTA-^†^cgcaccgcagtttggtcaat-3’ | |
| c.35G>T (p.G12V) | 5’-tgccctattgttgcgtcgga-3’ | 5’-^*^AGCTG**T**TGGCG-^†^tccgacgcaacaatagggca-3’ | |
| c.38G>A (p.G13D) | 5’-ctcatcggaagggctcgtaa -3’ | 5’-^*^CTGGTG**A**CGTAGG-^†^ttacgagcccttccgatgag-3’ | |
| Stabilizer sequence |  | | 5'-gcaagagtgccttgacgatacagctattcag-3’ |
| **Exon 3 Codon61**  **(Amino acid change)** | **Spotted capture probes^1^** | **Reporter sequences** | |
| Wild-type | 5’-gcctcgggcaaacgactaaa-3’ | 5'-^*^AGGTCAAGAGGAG-^†^tttagtcgtttgcccgaggc-3’ | |
| c.183A>C (Q61H*) | 5’-caccgacgctaatagttaag-3’ | 5'-^*^GTCA**C**GAGGAGTA-^†^cttaactattagcgtcggtg-3’ | |
| c.183A>T (Q61H*) | 5’-catacgcggtaaggatatag-3’ | 5'-^*^AGGTCA**T**GAGGAG-^†^ctatatccttaccgcgtatg-3’ | |
| c.182A>T (Q61L) | 5’-aatgctcgggaaggctactc-3’ | 5'-^*^GGTC**T**AGAGGAGTA-^†^gagtagccttcccgagcatt-3’ | |
| c.182A>G (Q61R) | 5’-tcttgacggaaaggtagaca-3’ | 5'-^*^AGGTC**G**AGAGGA-^†^tgtctacctttccgtcaaga-3’ | |
| c.181C>A ( Q61K) | 5’-atcccgtgagtcgatggttt-3’ | 5'-^*^AGGT**A**AAGAGGAGTA-^†^aaaccatcgactcacgggat-3’ | |
| Stabilizer sequence |  | | 5'-gagaaacctgtctcttggatattctcgacacag-3' |
| **Exon 4 Codon146**  **(Amino acid change)** | **Spotted capture probes^1^** | **Reporter sequences** | |
| Wild-type | 5’-cgcaccgcagtttggtcaat-3’ | 5'-^*^CATCAGCAAAGACA-^†^attgaccaaactgcggtgcg-3’ | |
| c.436G>A (A146T) | 5’-cacgcggcagtcgagttaat-3’ | 5'-^*^ACATCA**A**CAAAGACA-^†^attaactcgactgccgcgtg-3' | |
| Stabilizer sequence |  | | 5'- gctcaggacttagcaagaagttatggaattcctttta-3' |
| **NRAS Mutations** | | | |
| **Exon 2 Codon12-13**  **(Amino acid change)** | **Spotted capture probes^1^** | **Reporter sequences** | |
| Wild-type | 5’-cgagcacttaacattagagc-3’ | 5'-^*^AGCAGGTGGTG-^†^gctctaatgttaagtgctcg-3’ | |
| c.35G>C (p.G12A) | 5’-tccgaccttcgatctgtggt-3’ | 5'-^*^AGCAG**C**TGGTG-^†^accacagatcgaaggtcgga-3' | |
| c.34G>T (p.G12C) | 5’-atcgtacttggcactggagt-3’ | 5'-^*^GAGCA**T**GTGGTG-^†^actccagtgccaagtacgat-3' | |
| c.35G>A (p.G12D) | 5’-cgccgtatatggtcattggt-3’ | 5'-^*^AGCAG**A**TGGTGTT-^†^accaatgaccatatacggcg-3' | |
| c.34G>A (p.G12S) | 5’-gcccaaataagacgtgagcc-3’ | 5'-^*^GAGCA**A**GTG GTG-^†^ggctcacgtcttatttgggc-3' | |
| c.35G>T (p.G12V) | 5’-actcaaacataactctggcg-3’ | 5'-^*^AGCAG**T**TGGTGT-^†^cgccagagttatgtttgagt-3' | |
| c.38G>A (p.G13D) | 5’-acgagcgcataccatcgaag-3’ | 5'-^*^AGCAGGTG**A**TGTT-^†^cttcgatggtatgcgctcgt-3' | |
| c.37G>C (p.G13R) | 5’- tccgacgcaacaatagggca-3’ | 5'-^*^GCAGGT**C**GTGT-^†^tgccctattgttgcgtcgga-3' | |
| c.38G>T (p.G13V) | 5’-cctgctcgacaactagaaga-3’ | 5'-^*^AGCAGGTG**T**TGT-^†^tcttctagttgtcgagcagg-3' | |
| Stabilizer sequence |  | | 5'-ctgacaatccagctaatccagaaccactttgta -3' |
| **BRAF Mutation** | | | |
| **Exon 15 Codon600**  **(Amino acid change)** | **Spotted capture probes^1^** | **Reporter sequences** | |
| Wild-type | 5’-agcccggtctcatcgttgtt-3’ | 5'-^*^GCTACAGTGAAATCT-^†^aacaacgatgagaccgggct-3’ | |
| c.1799T>A (V600E) | 5’-agggatatgatacgtgcctt-3’ | 5'-^*^GCTACAG**A**GAAATCT-^†^aaggcacgtatcatatccct-3' | |
| Stabilizer sequence1 |  | | 5'-cgatggagtgggtcccatcagtttgaa-3' |
| Stabilizer sequence2 |  | | 5'-gaagacctcacagtaaaaataggtgattttggtcta-3' |
| Universal-Cy3^2^ | 5’-ctcaatgttcggactcag-3’ | | |

^1^ The spotted capture probes are amino modified in 5’-end; ^*^sequences which hybridize to single strand PCR (the variant base for each mutation is in bold and underlined); ^†^ the tails of the reporter oligonucleotides which hybridize to spotted capture probes. ^2^ The universal-Cy3 is labeled with Cyanine 3 in 3’-end.
